# Molecular Targets of Antihypertensive Peptides: Understanding the Mechanisms of Action Based on the Pathophysiology of Hypertension

**DOI:** 10.3390/ijms16010256

**Published:** 2014-12-24

**Authors:** Kaustav Majumder, Jianping Wu

**Affiliations:** 1 Department of Agricultural, Food and Nutritional Science, Faculty of Agricultural, Life and Environmental Sciences, University of Alberta, Edmonton, AB T6G 2P5, Canada; E-Mail: kaustav@ualberta.ca; 2 Cardiovascular Research Centre, Faculty of Medicine & Dentistry, University of Alberta, Edmonton, AB T6G 2S2, Canada

**Keywords:** antihypertensive peptides, spontaneously hypertensive rats, hypertension, angiotensin converting enzyme, endothelin, nitric oxide

## Abstract

There is growing interest in using functional foods or nutraceuticals for the prevention and treatment of hypertension or high blood pressure. Although numerous preventive and therapeutic pharmacological interventions are available on the market, unfortunately, many patients still suffer from poorly controlled hypertension. Furthermore, most pharmacological drugs, such as inhibitors of angiotensin-I converting enzyme (ACE), are often associated with significant adverse effects. Many bioactive food compounds have been characterized over the past decades that may contribute to the management of hypertension; for example, bioactive peptides derived from various food proteins with antihypertensive properties have gained a great deal of attention. Some of these peptides have exhibited potent *in vivo* antihypertensive activity in both animal models and human clinical trials. This review provides an overview about the complex pathophysiology of hypertension and demonstrates the potential roles of food derived bioactive peptides as viable interventions targeting specific pathways involved in this disease process. This review offers a comprehensive guide for understanding and utilizing the molecular mechanisms of antihypertensive actions of food protein derived peptides.

## 1. Introduction

Cardiovascular diseases account for approximately one third of the total deaths, totaling ~17 million annually worldwide [1]. Hypertension, the persistent elevation of blood pressure over 140/90 mm Hg (systolic/diastolic blood pressure, respectively), is considered one of the key risk factors for the development of cardiovascular diseases (CVD). Hypertension is often termed as “silent killer” affecting 1 billion people worldwide, and causes up to 9 million deaths every year [1]. In Canada, almost 6 million people (about 1 in every 5 adults), are affected by this condition [2]. Hypertension rarely presents with early symptoms, and even if diagnosed early, it is often treated inadequately [3,4]. Nevertheless, hypertension is a significant risk factor for atherosclerosis and hence predisposes to coronary heart disease, cerebrovascular disease, and renal disease [5,6,7,8,9]. In addition to tremendous health burden, treatment and prevention of hypertension are also associated with substantial socioeconomic consequences. The estimated costs for treating hypertension and related diseases were $156 billion in the USA in 2011 [10]. Pharmacological anti-hypertensive drugs are often associated with significant adverse side effects such as headache, dry cough, *etc.* [11,12]. Consequently, many patients still have their blood pressure poorly controlled and remain at increased risk for its complications even when treated with existing drugs [13,14]. Therefore, novel, cost-effective and efficient therapeutic strategies are urgently required for better management of hypertension.

It is well recognized that diet plays an important role in human health. Epidemiological studies have suggested that food habit or dietary choice can affect the prevalence of chronic diseases such as cardiovascular disease, obesity, and diabetes [15,16,17]. Diet manipulation studies such as dietary approaches to stop hypertension (DASH) suggest that adoption of a healthy diet (rich in fruits and vegetables) could lower high blood pressure [18,19]. Similarly, compounds like dietary sodium (present in table salt) and dietary potassium also have a great impact on blood pressure and associated vascular diseases [20,21,22]. Moreover, various clinical studies have demonstrated that macronutrients (protein, fat, and carbohydrate) can play key role in the management of high blood pressure. The optimal macronutrient intake to prevent heart disease (OmniHeart) trials demonstrated that partial replacement of carbohydrate with either protein or with monounsaturated fat could reduce high blood pressure, and the risk of coronary heart disease [23,24,25]. Indeed, food proteins also contain active peptide fragments encrypted within their structure that can exert beneficial effects on human health above and beyond their expected nutritional value. These active peptide fragments, known as bioactive peptides, can be released from their parent proteins by gastrointestinal digestion, fermentation, or food processing [26]. Food derived bioactive peptides have vast potential for applications as functional foods and nutraceuticals for the prevention and management of hypertension.

Among many types of food derived bioactive peptides, peptides with antihypertensive activity have received the most significant attention due to the persistence of hypertension and its associated complications even with pharmacological interventions [27,28,29]. These peptides target mainly at inhibiting angiotensin I converting enzyme (ACE), an enzyme playing a crucial role through renin angiotensin system (RAS) for the regulation of blood pressure and electrolyte balance in human body [7,30,31]. Peptides with anti-oxidant, anti-inflammatory, opioid receptor binding activities might also exhibit anti-hypertensive activity [32,33]. However correlation between *in vitro* and *in vivo* antihypertensive activities appears to be weak [29,32,34,35,36,37,38]. To develop effective antihypertensive peptides, it is important to understand the complex pathophysiology of hypertension and the potential targets where these bioactive peptides may exert their specific antihypertensive actions. The potential mechanisms of action of many food-derived peptides with antihypertensive activity have been previously reviewed [28,29,39,40,41,42]. However, limited information is available regarding the multiple functional roles of these peptides on various pathways involved in developing persistent hypertension.

Therefore, this particular review provides an overview about the complex pathophysiology of hypertension and highlights potential molecular targets of food derived peptides that may mediate the *in vivo* antihypertensive effects. Identification of these molecular targets can facilitate the use of food derived bioactive peptides as a novel therapeutics for the prevention and management of hypertension.

## 2. Pathophysiology of Hypertension

Hypertension develops from a complex interaction of genetic and environmental factors although more than 90% of cases do not have a clear etiology [43,44]. Previous research has identified major contributing factors: (i) increased sympathetic nervous system activity; (ii) increased levels of long term high sodium intake, inadequate dietary intake of potassium and calcium; (iii) altered renin secretion related to elevated activity of the RAS; (iv) increased activity of ACE resulting over production of angiotensin II (Ang II) and deactivation of kallikrein kinin-system (KKS); (v) endothelial dysfunctions and deficiencies of vasodilators including reduced nitric oxide (NO) bioavailability; (vi) abnormalities in vessel resistance due to vascular inflammation, increased activity of vascular growth factors and altered cellular ion channel [45,46,47,48,49]. Although all of the above factors clearly contribute to the pathogenesis of hypertension, the hyperactivity of the RAS, endothelial dysfunction, enhanced activation of sympathetic nervous system and structural abnormalities in resistance vessels play critical roles in the development and progression of this disease [49,50,51].

### 2.1. Renin Angiotensin System (RAS)

Physiologically, RAS is one of the important pathways for regulating blood pressure and vascular tone in human body [52,53]. The RAS pathway is initiated in the kidney with the proteolytic conversion of angiotensinogen to angiotensin I (Ang I) by renin. Ang I is an inactive decapeptide which can be converted into a vasoconstrictive octapeptide, Ang II, by the action of ACE. Ang II can be further cleaved by angiotensin converting enzyme 2 (ACE-2), to form angiotensin 1–7 (Ang_1–7_), then the G-protein-coupled receptor (GPCR)-Mas acts as an Ang_1–7_ receptor and initiates a counter-regulatory role by opposing Ang II induced vasoconstriction [7,54]. In addition, ACE-2 can also cleave a single amino acid from Ang I, producing inactive angiotensin 1–9 (Ang_1–9_). Ang II is an important regulator of fluid and sodium balance and also participates in cellular growth and remodeling [52,55].

**Figure 1:**
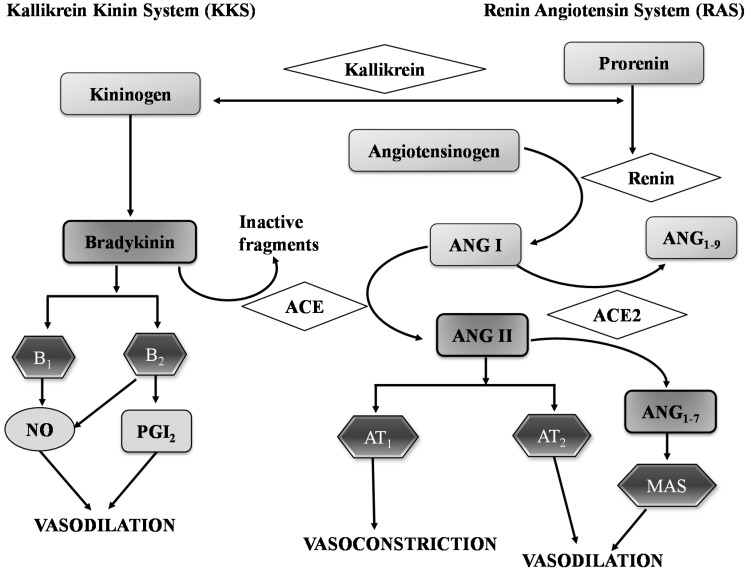
Renin-angiotensin system and kallikrein kinin system to regulate of blood pressure. Angiotensin I (Ang I), Angiotensin II (Ang II), Angiotensin converting enzyme (ACE), Angiotensin converting enzyme 2 (ACE 2), Angiotensin receptor 1 (AT_1_), Angiotensin receptor 2 (AT_2_), Bradykinin receptor 1 (B_1_), Bradykinin receptor 2 (B_2_), Nitric oxide (NO), Prostaglandins 2 (PgI_2_). Figure 1 modified from [63].

Ang II acts through two main receptors, angiotensin type 1 (AT_1_) and type 2 (AT_2_) receptors [30,53] (Figure 1). Binding to AT_1_ receptor causes vasoconstriction in vascular smooth muscle cells (VSMC). It also stimulates release of aldosterone to increase water and salt retention in the kidney, hypertropic growth of cardiomyocytes, and collagen synthesis of cardiac fibroblasts resulting in cardiac remodeling. In pathogenic conditions involving tissue remodeling and vascular inflammation, AT_1_ receptor is up regulated [56,57,58]. On the other hand, AT_2_ receptor presents in both endothelial and VSMC mediates vasodilation upon activation, releases NO, and inhibits cell growth [59]. Therefore, AT_1_ receptor mediates actions with potentially harmful consequences, whereas AT_2_ receptor, mediated actions exhibits protective effects against hypertension [53,60] (Figure 1).

Alternatively, ACE also actively participates in the KKS. Activation of ACE inactivates bradykinin, a potent vasodilator. Bradykinin acts through two different receptors, type 1 (B_1_) and type 2 (B_2_). Both receptors induce NO generation in endothelial cells [61,62]. In addition, B_2_ receptors also activate phospholipase A2 that releases arachidonic acid, which leads to the formation of several vasodilators including prostacyclin [30,31,53,63].

Though RAS is widespread in the body, the main source of renin is the juxtaglomerular apparatus of the kidney, while that of ACE is abundantly present cell surface of endothelial cells, especially in the lungs [64,65]. However, there is increasing evidence supporting an important role of local RAS, such as in the microvasculature of kidney, heart, and arterial tree, in the regulation of blood pressure [30,55,66].

### 2.2. Endothelial Dysfunction

Endothelial cells (EC) play important physiological functions in regulation of the vascular homeostasis or vascular balance under normal conditions. Impairment of normal vasorelaxant EC responses results in endothelium dysfunction. Endothelial dysfunction often disturbs the vascular function and creates a vascular imbalance that is responsible for various cardiovascular diseases including hypertension. Endothelial cells produce a number of vasoactive substances, including NO and endothelin (ET-1). NO the key vasodilator, and ET-1 a potent vasoconstrictor, are vital mediators of endothelial functions. An imbalance between these two factors is a feature of endothelial dysfunction.

**Figure 2:**
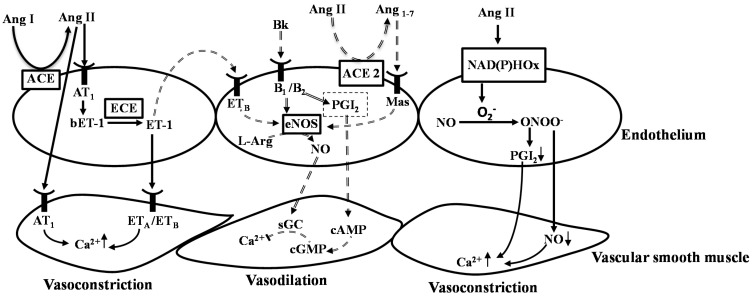
Endothelial dysfunction and blood pressure regulation. Angiotensin converting enzyme (ACE) converts angiotensin I (Ang I) to angiotensin II (Ang II), Ang II binds with angiotensin receptor 1 (AT_1_) on endothelium cells as well as vascular smooth muscle cells, then AT_1_ receptor increases calcium ion (Ca^2+^) concentration in vascular smooth muscle cells (VSMC) and exerts vasoconstriction. In endothelium cells activation of AT_1_ receptor increases the production of bET-1 (big endothelin-1). Endothelin-Converting Enzyme (ECE) converts bET-1 to endothelin-1 (ET-1) and exerts vasoconstriction by activating endothelin A/B receptors (ET_A/B_) in the VSMC. In contrast, activation of ET_B_ receptor in endothelium cells mediates vasodilatory effects via release of nitric oxide (NO) by activating endothelial nitric oxide synthase (eNOS). ACE also converts Bradykinin (Bk) into inactive peptides. Bk binds with bradykinin receptor (B_1/2_) and activates eNOS, which converts l-Arginine to l-Citrulline and produces NO. NO exerts vasodilation by activating cyclic guanosine monophosphate (cGMP) by inhibiting the concentration of Ca^2+^ in VSM. In endothelium cells Ang II produces superoxide (O_2_^−^) which scavenges NO and produces peroxynitrite (ONOO^−^), exerts vasoconstriction effect by limiting the supply of NO to the VSM. Signaling pathways illustrated with solid line arrows are representing vasoconstriction and with compound line arrows are representation vasodilation network. Figure 2 modified from [71].

NO initiates and maintains vasodilation through a cascade of biological events after diffusing through cell membrane [59]. NO is generated in endothelial cells by nitric oxide synthase (NOS) in a two-step five-electron oxidation of the terminal guanidine nitrogen of l-arginine, generating l-citrulline as a by-product. Three isoforms of NOS have been characterized: endothelial NOS (eNOS), neuronal NOS (nNOS) and inducible NOS (iNOS) [67]. Both eNOS and nNOS are present in the normal vascular endothelium [68,69]. After diffusion from endothelial to vascular smooth muscle cells, NO causes vasodilation [69] primarily by activating soluble guanylyl cyclase (sGC) and increasing intracellular concentration of cyclic guanosine-monophosphate [70] (Figure 2). Acute NOS inhibition results in vasoconstriction and reduction in peripheral blood flow [64]. These hemodynamic alterations are entirely reversible with administration of NO donors, such as glyceryl trinitrate (GTN) or sodium nitroprusside (SNP) [71], suggesting that the continuous presence of NO is required to prevent vasoconstriction. In addition, NO also affects cell metabolism, and inhibits mitochondrial respiration and ATP synthesis [59].

It has been suggested that NO bioavailability can be reduced in the presence of excessive reactive oxygen species (ROS) such as superoxide anion (O_2_^−^). Ang II enhances the formation of superoxide in endothelial cells by activating nicotinamide adenine dinucleotide phosphate (NADPH) oxidase. Superoxide readily reacts with NO to form peroxynitrite (ONOO^−^). Peroxynitrite is a strong oxidant, causing damage to cell membrane while leads to cell death and/or inflammation [72] (Figure 2). Excessive formation of O_2_^−^ also modifies tetrahydrobiopterin (BH_4_) a cofactor for NOS [64]. In the absence of this cofactor, NOS can become uncoupled and paradoxically generated O_2_^−^ instead of NO [58]. Decreased bioactivity of NO could switch the cellular signaling from NO-mediated cellular processes to oxidant-mediated redox signaling, stimulating pro-inflammatory pathways, and ultimately leading to vascular remodeling and resulting in increased blood pressure [73].

In contrast to NO, circulating endothelins have vasoconstrictory properties. Three isoforms for endothelins (ET-1, ET-2, and ET-3) have been characterized but ET-1 is the dominant form and actively modulates vascular functions [74]. ET-1 is synthesized predominantly in endothelial cells and also in vascular smooth muscle cells [74]. Its precursor, preproET-1 (ppET-1) is a functionally inactive peptide which is sequentially cleaved by cellular enzymes and ultimately produces the vasoactive ET-1. Furin-like proteases cleave ppET-1 to generate a 39 amino acid peptide (38 amino acids in humans) called big-ET-1 (bET-1). Under normal physiological conditions, endothelin-converting enzyme (ECE) converts big endothelin (bET-1) to ET-1, whereas current evidence suggests ET-1 can be produced from bET-1 through several other proteolytic digestions involving matrix metalloproteinases (MMPs), and neutral endopeptidase (NEP). ET-1 exerts its functions by binding to G protein-coupled ET receptors, endothelin receptor A (ET_A_) and endothelin receptor B [75]. ET_A_ receptors are located within the VSMC, whereas ET_B_ receptors are located both on vascular endothelium, as well as, on VSMC. Binding with ET_A_ and ET_B_ receptors in vascular smooth muscle ET-1 exerts vasoconstriction. Alternatively, ET-1 binding to ETB receptors in the endothelium results in vasodilation through increased NO and prostacyclin synthesis [74]. The interplay between NO and ET-1 is important in numerous pathophysiological conditions [76]. The reduction in NO bioavailability is associated with increased ET-1 expression. Similarly, NO antagonizes the ET-1 pathway via several different mechanisms [75]. These relationships suggest an intimate link between these two mediators to maintain a delicate balance in endothelial function [74]. ET-1 also stimulates the release of pro-inflammatory cytokine such as interleukin (IL)-1, and IL-8. Factors like Ang II, thrombin, and inflammatory cytokines (tumor necrosis factor-α, IL-1, IL-2) can modulate the expression of ET-1 in endothelial cells by enhancing the gene expression of ppET-1. Therefore reduced bioavailability of NO and excessive production of Ang II can directly induce endothelial dysfunction and subsequent increase in blood pressure.

### 2.3. Sympathetic Nervous System

The sympathetic nervous system is a part of the autonomic nervous response system that can be activated by environmental stress. Increased sympathetic nervous system activity can cause both arteriolar constriction and arteriolar dilation [77]. Thus, the autonomous nervous system contributes to the development and maintenance of hypertension through stimulation of cardiac output in heart, fluid retention in kidney and increased vascular resistance in peripheral vasculature [77].

**Figure 3:**
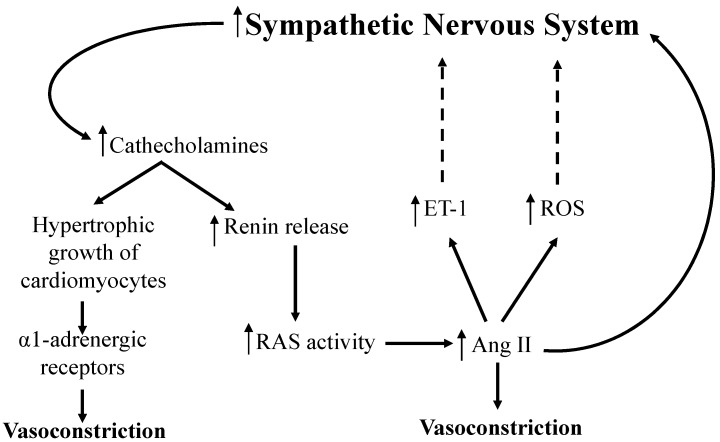
Regulation of blood pressure through autonomic nervous system. Increased sympathetic nervous system stimulates the release of cathecholamines from post ganglionic neurons. Cathecholamines increases the hypertrophic growth of cardiomyocytes and release more renin in adrenal cortex. Increase production renin over activates renin angiotensin system (RAS) and produces more Angiotensin-II (Ang II). Hypertrophic growth of cardiomyocytes and increase production of Ang II results in vasoconstriction. In addition, Ang II production increases ET-1 (Endothelin-1) and ROS (reactive oxygen species) production and directly affect the over activity of sympathetic nervous system.

Sympathetic nervous system stimulates the release of catecholamines (norepinephrine and epinephrine) from postganglionic neurons [78]. The release of catecholamines activates the hypertrophic growth of cardiomyocytes [79]. Simultaneously, catecholamine release increases the activity of β-adrenoceptors while decreases the activity of α-adrenoceptors, which in turn results in the conversion of pro-renin to the active form of renin [79]. The release of renin subsequently activates RAS and results in increased blood pressure through the production of Ang II (Figure 3). Ang IAng II also amplifies the response of the sympathetic nervous system by a peripheral mechanism, that is, pre-synaptic facilitatory modulation of norepinephrine release [77,78]. Additionally, ROS and ET-1 may also stimulate the sympathetic activity and its effects on the vasculature [78,80]. Thus increased sympathetic activity is associated with the development of hypertension.

### 2.4. Vascular Remodeling

Vascular remodeling contributes to increased peripheral resistance, alterations in vessel structures, development of hypertension, and the consequent end organ damage during hypertension [6,48]. Hypertension associated with structural changes in the vessels has been called as remodeling. Vascular remodeling is an active process and involves changes in cellular processes, such as cell growth, cell migration, cell death, and degradation/synthesis of extracellular matrix [48,81]. Remodeling of vessels increases peripheral resistance in pre-capillary vessels including arterioles and small arteries (lumen diameters < 300 µm). These structural changes of the vessels reduce the lumen diameter and increase the media-to-lumen ratio (M/L) and ultimately, increase both vascular reactivity and peripheral resistance [48].

Vascular inflammation can induce endothelial dysfunction, which ultimately results in vascular remodeling. Under resting conditions, EC prevent leukocyte adhesion but local overproduction of Ang II can initiate the expression of adhesion molecules on endothelial cells which results in adhesion of leukocyte to the inner arterial wall in a stepwise manner, known as leukocyte recruitment [82]. Subsequently Ang II can also induce oxidative stress resulting in excessive production of ROS. ROS, in turn, stimulates the production of cytokines such as tumor necrosis factor-α (TNF-α), IL-1β *etc.* [83]. TNF-α activates the phosphorylation of nuclear transcription factor-κB (NF-κB), which leads to the expression of adhesion molecules (ICAM-1, intercellular adhesion molecule-1; VCAM-1, vascular cell adhesion molecule-1), and the release of monocyte chemotactic protein-1 (MCP-1). The over-expression, of these molecules then recruits the leukocytes (monocytes and macrophages) to the site of inflammation [82]. Over-expressed inflammatory response together with the oxidized lipid molecules forms plaques in the interstitial space between endothelial and vascular smooth muscle. Formation of plaque directly contributes to vascular remodeling, increases blood pressure and initiates atherosclerosis.

Matrix metalloproteinases (MMP) are zinc (Zn) and calcium (Ca) dependent proteolytic enzymes that degrade extracellular matrix proteins [84]. Several different MMPs are present in the vasculature and their synthesis is induced by cytokines and cell to cell-matrix interactions. An increasing body of scientific evidence demonstrates that uncontrolled proteolytic process is one of the key mechanisms for the development of hypertension and MMP play a crucial role in this process [84,85]. In Ang II-induced hypertension, MMP are responsible for elevated blood pressure and tissue fibrosis [85]. Acute release of MMP2 cleaves the sarcomere proteins (titin, troponin I and myosin light chain-I) that can impair cardiomyocyte contractility [86,87]. Similarly, MMP-7 is one of the major inducing factors for endothelial dysfunction. MMP-7 also promotes GPCR agonist (*i.e.*, Ang II) induced vasoconstriction through epidermal growth factors (EGF) and subsequently increases the blood pressure and cardiovascular hypertrophy [88]. Thus MMP with accessory signaling molecules can modulate cell-cell interaction, release of cytokine, and chemokines, which ultimately propagate the vascular inflammatory response.

**Figure 4:**
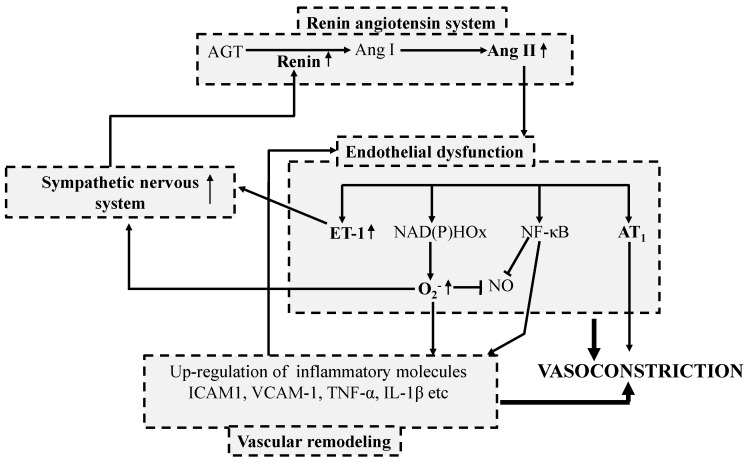
Pathophysiology of hypertension—a vicious cycle. Renin angiotensin system (RAS), endothelial dysfunction, vascular remodeling, and activity of sympathetic nervous system are correlated with each other. Enhanced RAS activity leads to over production of angiotensin II (Ang II) which accelerates endothelial dysfunction. Ang II induced endothelial dysfunction results in vasoconstriction as well up regulates the activity of transcription factors (such as NF-κB, nuclear factor κB), promoting vascular inflammation. Vascular inflammation up regulates the expression of leukocyte adhesion molecules such as ICAM-1 (Intercellular adhesion molecule 1), VCAM-1 (Vascular adhesion molecule-1) as well as inflammatory cytokines like TNF-α (Tumor necrosis factor-α) and IL-1β (Interleukin-1β). Similarly, during endothelial dysfunction over expression of ET-1 (Endothelin-1) and increased levels of ROS such as superoxide (O_2_^−^) can directly increase the sympathetic nervous system. Finally, increased sympathetic nervous system increases renin production which eventually activates RAS.

Apart from the pathways described above, there are other factors such as sodium/water excretion, adrenal steroids, *etc.* that also contribute to the development of high blood pressure. Interestingly, one or more component of each distinctive pathway can modulate or activate another pathway and thus create a complex cycle for the development of hypertension (Figure 4). The pathophysiologic mechanisms that lead to blood pressure elevation are so complex that anti-hypertensive treatment based on a single pathway is rarely feasible (ACE inhibitors may be an exception, although Ang II involves both RAS and pro-inflammatory pathways); [89]. Current pharmacological treatment approaches for treating hypertension are very much selective, which may explain the inadequacy in palliation of hypertension and adverse side effects. Food protein-derived peptides have been widely studied for controlling elevated blood pressure, but it is essential to understand their effects on the pathogenic mechanisms and the interplay between different molecules to develop these as novel therapeutic agents.

## 3. Antihypertensive Peptides from Food Proteins—Mechanisms of Action

Food protein-derived peptides exhibit antihypertensive effects through various mechanisms. A majority of food protein-derived antihypertensive peptides have been initially identified as ACE inhibitors using *in vitro* methods. Peptides with ACE inhibitory properties were isolated first from snake (*Bothrops jararaca*) venom [90,91]. This work encouraged several subsequent investigations to look for food protein derived peptides as antihypertensive alternatives. Earlier studies have identified several ACE-inhibiting peptides from both plant (especially soybean) and animal sources (milk, fish and egg proteins) [27,92,93,94,95]. Given the complexity of blood pressure regulation, it is important to understand the mechanism of action of a peptide in order to develop functional foods or nutraceuticals for the prevention and management of hypertension. The following sub-sections briefly describe the effect of food-derived bioactive peptides on modulating RAS function, ameliorating endothelial dysfunction, modulating sympathetic nervous system, and vascular inflammation.

### 3.1. Antihypertensive Peptides Modulating RAS Function

ACE inhibition is the main mechanism by which peptides can modulate RAS function and exert antihypertensive effects. A number of *in vivo* studies performed in animals and/or humans have demonstrated that various food-derived peptides could significantly reduce blood pressure through ACE inhibition upon either intravenous or oral administration [93,94,95,96,97,98,99,100]. Milk protein derived peptides are known for their antihypertensive activity. The release of antihypertensive peptides from milk protein has been achieved through two different approaches: hydrolysis of milk protein by proteolytic enzymes and fermentation of milk. One of the first peptides identified from tryptic digestion of α_s1_-casein, FFVAPFPGVFGK, could significantly reduce both systolic blood pressure (SBP by 34 mmHg) and plasma ACE activity at a dose of 100 mg/kg BW in spontaneously hypertensive rats [38]. MKP, another peptide identified from the tryptic digest of bovine casein has also shown antihypertensive effect in SHRs. The crude hydrolysate containing only 0.053% of MKP significantly reduced the SBP by 40 mmHg at a dose of 100 mg/kg BW 2 h after administration, whereas the purified peptide MKP exhibited a maximum SBP reduction of 45 mmHg 8 h after administration in SHR. Both preparations also exhibited ACE inhibitory properties [101]. Three peptides, IAK, YAKPVA, and WQVLPNAVPAK from α_s1_-casein produced by combined action of pepsin, chymotrypsin, and trypsin showed a significant decrease in both SBP and diastolic blood pressure (DBP) in SHR with doses of 4, 6, and 7 mg/kg BW, respectively [99]. The authors determined that the ACE inhibitory property of these peptides was responsible for the observed antihypertensive effect [99]. Two tripeptides, VPP and IPP, produced from milk fermentation with a combination of *Lactobacillus helveticus* and *Saccharomyces cerevisiae*, were the well-known antihypertensive peptides from milk [102,103]. Single oral administration of VPP and IPP at a dose of 0.6 and 0.3 mg/Kg BW could significantly reduce SBP by 32 and 28 mmHg, respectively [104]. SHRs fed with fermented milk containing these peptides demonstrate significant decreases in serum ACE activity and BP [104].

Apart from milk, fish protein derived peptides have also been shown to exhibit antihypertensive effect through ACE inhibition. Three peptides LKP, IKP, and IWH identified from hydrolysate of dried bonito have been shown to significantly reduce SBP in SHR animals [29,105]. Another peptide LKPNM, also identified from bonito hydrolysate, was found to exert a longer-term effect on SHRs than LKP. The authors identified LKPNM as a “pro-drug” type ACE inhibitor, which could serve as a precursor to the actual ACE inhibitor released upon gastrointestinal proteolysis [105].

Egg protein ovalbumin derived peptide YPI reduces blood pressure by 30 mm Hg after a single oral administration to SHRs and its actions were likely mediated through ACE inhibition [94]. A study from our own research group identified a potent ACE inhibitory tri-peptide IRW from thermolysin-pepsin hydrolysate of egg white protein ovotransferrin [106]. In SHRs, IRW significantly reduced SBP by 40 mmHg after 18 days of treatment at a dose of 15 mg/kg BW while concomitantly decreased plasma Ang II levels, likely through ACE inhibition [97].

AT_1_, the Ang II receptor, is one of the targets to modulate increased RAS activity. In addition to inhibition of ACE, AT_1_ receptor blockade is considered as an effective therapy for hypertensive patients [107]. Moreover, it is a useful alternative approach for the patients sensitive to side effects of ACE inhibitors [107]. Similarly, renin is a key regulator of RAS; therefore inhibition of renin could also alter RAS activation. Milk lactoferrin derived peptides RRWQWR, LIWKL, and RPYL significantly reduced blood pressure in SHRs and were also found to reduce Ang II induced vasoconstriction in isolated rabbit carotid arterial segments [108]. Among these three peptides, RPYL showed the maximum effect, and demonstrated inhibition of Ang II binding to AT_1_ receptors [109]. Recently, egg protein derived peptide RVPSL has been shown to significantly decrease SBP by 25 mmHg after 4 weeks of treatment at a dose of 50 mg/Kg BW. The mRNA levels of renin, ACE, and AT_1_ receptor in kidney and serum level of AngII and renin were all significantly decreased by RVPSL treatment [109]. Results from these studies suggest that food derived bioactive peptides can indeed act upon the AT_1_ receptor and/or act as a renin or ACE inhibitors to exert their *in vivo* antihypertensive effects [109]. Likewise, renin inhibition is also a crucial mechanism of controlling blood pressure by reducing the formation of Ang II. Glycyl histidinyl serine (GHS), a peptide isolated from pepsin, pancreatin digestion of rapeseed protein exhibited both ACE and renin inhibitory activity and oral administration of this peptide (30 mg/kg BW) reduces blood pressure in SHR 6 h after administration [110].

Interestingly in a different study, Ehlers *et al.* had demonstrated that the vasorelaxation effect of IPP may be mediated though ACE-2, Ang_1–7_, and Mas axis. In an *ex vivo* experiment the authors demonstrated that the administration of IPP can produce more Ang_1–7_ and exert vasorelaxation activity on Mas receptor possibly through modulation of ACE-2 [111]. Similarly, study from our group has found that IRW treatment could increase the gene expression of ACE-2 in mesenteric artery (*unpublished data*), which may further convert Ang II to Ang_1–7_ and exerts vasodilation. Thus activation of ACE-2 through peptide treatment could exert beneficial effect for the prevention of hypertension. Peptides previously identified as ACE inhibitors though *in vitro* method could actually exhibit various effects on the RAS and thus reduce blood pressure.

### 3.2. Antihypertensive Peptides Ameliorating Endothelial Dysfunction

Increased production of vasoconstrictory/pro-inflammatory mediators like ET-1 and superoxide (O_2_^−^) decreases the bioactivity of vasodilatory NO resulting in endothelial dysfunction. Food protein derived antihypertensive peptides have been shown to improve endothelial functions and cause vasodilation. The three main mechanisms by which food derived antihypertensive peptides modulate endothelial function are increased production of vasodilatory factors (*i.e.*, NO and prostaglandins), reduced production of vasoconstriction factors (*i.e.*, ET-1), and increased anti-oxidant activity.

Egg protein ovalbumin derived peptide RADHP could significantly reduce blood pressure by 28 mmHg after a single oral administration in SHR animals [112]. RADHP also exhibited a dose-dependent relaxation in an isolated SHR mesenteric artery. However, the removal of endothelium from mesenteric artery was associated with disappearance of the relaxation effect, suggesting endothelium-dependent vasodilator activity of RADHP [113]. Moreover, pretreatment with *N*-nitro-l-arginine methyl ester (l-NAME), a NOS inhibitor, inhibited the RADHP mediated vasodilation; while pretreatment with superoxide dismutase (SOD), a radical scavenger, did not alter RADHPF induced vasodilation, suggesting that the vasodilatiory effect was unlikely to be caused by scavenging (O_2_^−^), but possibly resultant from stimulating NO production [114]. Four other peptides, YRGGLEPI, YR, ESI, and NF from egg protein ovalbumin have also demonstrated *in vivo* antihypertensive effect in SHRs [94,115]; their actual mechanisms of action remain unknown. However, it was evident that these antihypertensive effects were independent of ACE inhibition, as in an isolated mesenteric artery experiment vasorelaxation activity of these peptides were blocked by the treatment of NO inhibitor l-NAME and cyclooxygenase inhibitor indomethacin [29,115]. In addition to their ACE inhibitory effects, VPP and IPP also demonstrate the capability to improve vascular endothelial dysfunction. Yamaguchi *et al.* demonstrated that VPP and IPP administration could significantly increase the mRNA expression of eNOS in SHR [115]. Increased expression of eNOS directly correlates with enhanced production of NO and reduction of BP. A later study also showed that administration of VPP and IPP to cultured endothelial cells could significantly increase the NO production [116,117,118]. Results from these studies clearly indicate that antihypertensive peptides VPP and IPP could induce vasodilation through NO, independently of ACE inhibition. Results from our previous study also demonstrate that egg protein ovotransferrin derived peptide IRW treatment could increase the NO mediated vasodilation in mesenteric arteries of SHR animals, probably through increasing eNOS expression [97].

Increased bioavailability of NO can also improve vasodilation and reduce BP. Therefore, antioxidant and free radical scavenging activities could be beneficial for altering endothelial dysfunction. Milk-derived peptides RYLGY and AYFYPEL, obtained from bovine casein hydrolysate, have shown *in vivo* ACE inhibitory and anti-oxidant effects [118]. Oral administration of these peptides significantly reduced BP in SHR animals at a dosage of 5 mg/kg BW. The authors conclude that *in vitro* ACE inhibitory activity and radical scavenging activity of these peptides could potentially contribute towards reduction in blood pressure in SHR [118]. Therefore, the vasoprotective activity of these peptides could reduce blood pressure and potentially ameliorate the vascular fibrosis. Additionally, another peptide MY, derived from sardine muscle, exhibited antihypertensive effect by suppressing ROS generation in endothelial cells via induction of hemeoxygenase-1 (HO-1) and ferritin [119]. A study from our group has shown that the egg derived peptides IRW and IQW could significantly reduce TNF-α induced oxidative stress in cultured endothelial cells [120]. Moreover, oral administration of IRW could reduce oxidative stress in aorta and kidneys in intact SHR animals [97]. Thus, by acting as an anti-oxidant, these peptides play a crucial role to improve NO bioavailability and consequently modulate endothelial function and BP.

Apart from increased bioavailability of NO, treatment of bioactive peptide can also release various other vasodilatory factors such as prostaglandins (PGI_2_). Zhao *et al.* identified an antihypertensive peptide MRW from pepsin-pancreatin digest of spinach Rubisco (Ribulose bisphosphate carboxylase/oxygenase) protein. Oral administration of MRW at a dose of 5 mg/Kg BW could significantly reduce the SBP by 20 mmHg in 25 week old male SHR [121]. MRW also exhibited a dose-dependent vasodilation in an *ex vivo* study on isolated mesenteric arteries of SHRs; the relaxation effect of MRW was not NO-dependent, and was mediated by upregulation of PGI_2_ possibly through B_2_ receptor activation [121]. A similar effect was observed with RIY, a peptide derived from the rapeseed protein napin [122]. The study by Yamada *et al.* suggested that the antihypertensive effect of RIY in SHRs is induced mainly by the production of PGI_2_ [123].

The interplay between NO and ET-1 is well established in the context of endothelial dysfunction. The ECE plays an important role in converting inactive bET-1 to vasoactive ET-1, which subsequently binds to ET receptors and induces vasoconstriction. Therefore, ECE inhibitors or ET receptor agonists are the key targets for the antihypertensive therapy [124]. Bovine β-lactoglobulin derived peptide ALPMHIR was found to suppress the ET-1 activity in porcine aortic endothelial cells [125], possibly through ECE inhibition. In another study, eight peptides derived from lactoferricin B those were previously characterized as ACE inhibitors have showed significant inhibition of ECE activity when vasoconstriction was induced by big ET-1. Lfcin_17–25_ (FKCRRWQWR), LfcinB_17–31_ (FKCRRWQWRMKKLGA), LfcinB_17–32_ (FKCRRWQWRMKKLGAP), and Lfcin_19–25_ (CRRWQWR), were the most potent among them; these peptides were shown to inhibit ECE in isolated aortic segments from rabbits [126]. Furthermore, in a follow up study, the same group identified two more ECE inhibitory peptides, GILRPY and REPYFGY, from bovine lactoferrin hydrolysate [127]. Interestingly, these studies also suggested that these peptides may act either as dual vasopeptidase inhibitors (ACE/ECE), or as specific ECE inhibitors to produce their vasorelaxant effects [127]. So far there is little evidence to prove the relationship between ACE and ECE inhibition [127]; therefore these peptides may have dual enzyme inhibitory effect, which might result in pronounced blood pressure reducing effects.

Endothelial dysfunction leads to influx of calcium ion (Ca^2+^) in VSMC and increases vasoconstriction by activation of AT_1_ and ET_A_/ET_B_ receptors (Figure 2). Therefore, blocking of calcium channel reduces influx of Ca^2+^ and results in vasodilation. Peptides derived from fish protein hydrolysate have been shown antihypertensive effects by blocking the Ca^2+^ channels [128]. VY, a peptide derived from sardine muscle, exhibited an antihypertensive effect in SHRs as well as in Tsukuba-Hypertensive Mouse (THM) at doses of 10 and 0.1 mg/g BW respectively [129,130]. A study by Tanaka *et al.* showed that the antihypertensive effect of VY is actually mediated upon the VSM and it acts as an L-type Ca^2+^ channel blocker [128]. Similar mechanism has also been proposed for the sardine-derived peptide WH that suppresses the extracellular Ca^2+^ influx by blocking the L-type Ca^2+^ channel blocker in human VSM cells [131,132].

### 3.3. Antihypertensive Peptides Modulating Sympathetic Nervous System and Controlling Blood Pressure

Opioid receptors are present in the central nervous system and they are involved in the regulation of blood pressure through increasing the activity of the sympathetic nervous system [133]. A peptide (YGLF) derived from pepsin/trypsin digestion of α-lactorphin has been shown to reduce blood pressure in SHR by binding to opioid receptors [134]. The vasodilatory effect of YGLF is endothelial dependent and can be inhibited by selected eNOS inhibitors [135]. Therefore, a novel mechanism of binding to endothelial opioid receptors and subsequent NO release might be responsible for the vasodilatory effects of this peptide.

### 3.4. Antihypertensive Peptides Modulating Vascular Remodeling

Vascular inflammation-induced peripheral resistance is a contributor to elevated blood pressure and associated vascular pathologies [81]. Milk-derived tripeptide VPP pretreatment could significantly decrease phorbol 12-myristate 13-acetate (PMA) induced monocyte adhesion to human endothelial cells [136]. In addition, treatment with the both VPP and IPP offered protection against the development of atherosclerotic plaques in the apolipoprotein E (ApoE) knockout mice through a combined action involving the modulation of inflammatory as well as hypertensive pathways [137]. In our laboratory, the egg-derived tri-peptide IRW has demonstrated antihypertensive and anti-inflammatory properties by controlling both the hyperactive RAS pathway as well as the inflated pro-inflammatory cytokine levels in SHR [97]. Increased circulating levels of proinflammatory cytokines during the hypertension could further contribute to endothelial dysfunction and up-regulation of leukocyte adhesion in the vasculature, which ultimately results in vascular remodeling. Therefore, peptides that control the inflammatory pathways could potentially improve the vascular pathogenesis, and hence, control vascular remodeling and reduce elevated blood pressure.

**Figure 5:**
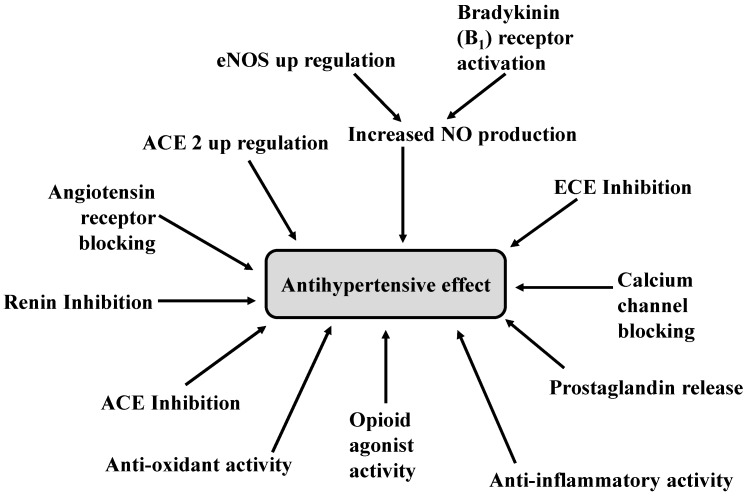
A schematic diagram of antihypertensive mechanism of food derived peptides. ACE (Angiotensin-I converting enzyme), ACE 2 (Angiotensin converting enzyme 2), eNOS (Endothelial nitric oxide synthase), NO (Nitric oxide), ECE (Endothelin converting enzyme).

To date, several food protein-derived antihypertensive peptides have been reported with a significant antihypertensive activity in animal studies, mostly with SHR (Table 1). Based on current available scientific evidences, it can be concluded that food protein-derived peptides may exert antihypertensive activity through multiple mechanistic pathways as follows: ACE inhibition, renin inhibition, ACE-2 activation, AT_1_ receptor blocking, increase NO production, ECE inhibition, PGI_2_ activation, blocking of Ca^2+^ channel, opioid activity, anti-oxidant activity and anti-inflammatory activity (Figure 5). Different peptide sequences have different modes of action, which may be mediated through their structural features. Various vasodilatory mechanisms of different peptides are summarized in Table 1.

**Table 1:** Antihypertensive activity and vasodilatory mechanism of food derived bioactive peptides in spontaneously hypertensive rats.

Vasodilatory Mechanism	Food	Protein	Peptide Sequence	Dose mg/kg BW	SBP Decrease (mm Hg)	References
ACE inhibition	Milk	α-casein	MKP	0.5	−30.0	[101]
		κ-casein	IAK	4.0	−20.7	[138]
			YAKPVA	6.0	−23.1	[139]
		β-casein	IPP	0.3	−28.3	[102,117]
			VPP	0.6	−32.1	[102,117]
	Egg	Ovotransferrin	IRW	15.0	−40.0	[97,106]
	Fish	Bonito muscle	LKP	2.25	−5.0	[105]
AT_1_ blocker	Egg	Egg white protein	RVPSL	50.0	−25.0	[109]
Ca^2+^ channel blocker	Fish	Sardine Muscle	VY	10.0	−18.5	[129]
PGI_2_ activator	Rapseed	Napin	RIY	7.5	−28.0	[123]
	Spinach	Rubisco	MRW	5.0	−20.0	[121]
Renin inhibition	Egg	Egg white protein	RVPSL	50.0	−25.0	[109]
ACE-2 activation	Milk	β-casein	IPP	0.3	−28.3	[111]
Anti-oxidant	Milk	α-casein	MKP	0.5	−30.0	[102]
			RYLGY	5.0	−32.0	[139]
			MY	10.0	−19.4	[39,119]
Opioid-agonist	Milk	α-lactorphin	YGLF	1.0	−23.7	[134]
eNOS up-regulation	Milk	β-casein	IPP	0.3	−28.3	[115]
			VPP	0.6	−32.1	[115]
	Egg	Ovotransferrin	IRW	15.0	−40.0	[97,106]

## 4. Antihypertensive Effects of Food Derived Peptides—Clinical Studies

Clinical trials are necessary to evaluate the efficacy of food protein derived bioactive peptides in humans. It is also important to study the pharmacokinetics for the development of nutraceuticals and/or functional foods from food protein derived bioactive antihypertensive peptides. Two well-known peptides, VPP and IPP have shown efficacy as antihypertensive agents in human clinical studies [139]. Oral administration of VPP and IPP incorporated in different food formulas (fermented milk, fruit juice) demonstrated significant decrease in blood pressure (SBP and DBP) in Japanese and Finnish hypertensive volunteers [140,141]. However, the oral intake of these same peptides failed to reduce BP in Dutch and Danish hypertensive subjects, suggesting possible differences in efficacy among different human populations [142]. A meta-analysis of 18 clinical trials has shown that oral administration of these peptides (VPP and IPP) does reduce BP in hypertensive subjects but the beneficial effect appears to be pronounced in Asian subjects [143]. The controversial results obtained by different studies about the effect of lactotripeptides in Caucasian population was addressed by Boelsma *et al.* in a double blinded placebo control trial with 70 Caucasian pre-hypertensive or stage-1 hypertensive subjects. The result from this study reveals that oral administration of IPP exerts clinically relevant BP reduction in Caucasian subjects with stage-1 hypertension [96]. Furthermore, another study has demonstrated that administration of milk tri-peptides along with plant sterols can exhibit a clinically significant reduction in SBP as well as serum total and low density lipoprotein (LDL)-cholesterol without adverse effects in hypertensive, hypercholesterolemic subjects in a randomized, placebo-controlled double-blind intervention [144]. Results from all these studies suggested that the reduction of blood pressure has been observed after at least 1–2 weeks of treatment with maximum effect of 13 mm Hg for SBP and 8 mm Hg for DBP with a dose range of approximately 3–55 mg/day. A recent meta-analysis showed that small doses (2.0–10.2 mg/day) of milk casein derived tri-peptides (VPP and IPP) exhibited an overall reduction of SBP by 4.0 mm Hg and DBP by 1.9 mm Hg in mildly hypertensive subjects [145]. Another clinical trial conducted by Hirota *et al.* with 24 mildly hypertensive subjects showed that these two lactopeptides (VPP and IPP) also improves endothelial dysfunction and significantly increase hyperemia measured on the left upper forearm of the subjects [146]. In addition to VPP and IPP, another study showed that consumption of yogurt enriched with casein derived antihypertensive peptides (RYLGY and AYFYPEL) reduced significantly SBP by 12 mmHg after 6 weeks of intake in a normalized placebo control trial [29]. Human clinical trials with pea protein hydrolysate also showed significant decrease in SBP by 5–7 mm Hg after 2 weeks treatment, but the smaller number (*n* = 7) used in this study is obviously not enough to judge the efficacy of the hydrolysate [147]. Similarly, sardine muscle derived di-peptide (VY) was used for human clinical trials. A randomized double-blind placebo control trials with 29 hypertensive subjects have demonstrated decrease in SBP and DBP by 9.3 and 5.2 mmHg, respectively, after 4 weeks of treatment [148]. However the clinical impact is controversial due to the small number of heterogeneous subjects in the trials [39]. Another trial involving 63 hypertensive subjects have shown that consumption of a vegetable drink containing VY could significantly reduce the blood pressure in high and mild hypertensive subjects, without any adverse side effects [149]. The third clinical study showed that a single oral administration of VY could significantly increase the plasma VY level, indicating the absorption of peptide in the blood stream. However no marked decrease in blood pressure was observed with the increased plasma VY level, indicating that VY did not exhibit an acute anti-hypertensive effect after oral ingestion and a longer-term treatment was likely necessary for the clinical benefits [150].

Various animal studies showed BP reduction by 20–40 mmHg using food protein derived bioactive peptides [29,99,115,118]; however, studies on human subjects were limited and the extent of blood pressure reduction was much less, mostly by 2–12 mmHg [142,143,151]. It should be noted that most animal studies were performed in a specific model of hypertension (such as SHR) where all animals had the same particular pathophysiology but the human subjects were likely to have different etiology underlying their clinical hypertension. In addition, animal studies use specific strains of animals while a group of human subjects in a clinical study are likely to have diverse racial and genetic backgrounds, which might complicate the efficacy of bioactive peptides. Indeed racial and genetic factors are known to modulate the development of hypertension [152,153]. Moreover, pharmacokinetics and pharmacodynamics of these food-derived bioactive peptides could be different in rodents and humans further complicating the efficacy of bioactive peptides. Therefore, more clinical studies engaging the volunteers from various ethnicities are required to establish the efficacy of food derived bioactive peptides as clinically relevant antihypertensive agents (Table 2).

**Table 2:** Human clinical trials of food protein derived antihypertensive peptides.

Active Peptide	Administered Product	Study Description	Dose/Day	Duration (Weeks)	SBP Decrease (mmHg)	References
VPP and IPP	Fermented milk	Double-blinded placebo-controlled randomized trial, 46 men with high-normal blood pressure.	150 mL (3.0 mg VPP and 2.25 mg IPP/100 g)	21	−5.2 mm Hg	[144]
	Evolus^®^ (fermented milk flavored with fruit juice)	Placebo-controlled randomized trial, 42 subjects with mild hypertension.	160 g	4	−6.7 mm Hg	[143]
	Low-fat yoghurt drinks	Randomized double-blind placebo-controlled trial, 135 hypertensive subjects (male/female: 88/47).	200 mL (5.8 mg VPP and 5.4 mg IPP)	8	No significant difference in blood pressure between the treatment and placebo controlled group	[145]
	Milk protein hydrolysate	Placebo control, double blinded, crossover including 70 Caucasian subjects.	2-tablets/day (each tablet contains 7.5 mg IPP)	4	−4.0 mm Hg in SBP (significant reduction) No change in DBP	[97]
	Fruit Juice fortified with Lacto tri-peptides	Randomized double blinded, 52 (men:women = 29:21) mildly hypertensive patients.	25 mL/day (3.0 mg of VPP and IPP)	6	−5.0 mm Hg in SBP	[154]
	A lacto spread contained VPP, IPP and plant sterols	Randomised, placebo-controlled double-blind intervention, 104 hypertensive, hypercholesterolemic subjects.	20 g/day (containing 4.2 mg of VPP and IPP; 2 g of plant sterols)	10	−4.1 mm Hg in SBP, No change in DBP and significantly reduce plasma LDL cholesterol	[147]
RYLGY and AYFYPEL)	Casein hydrolysate	Normalized placebo control trial.	20 mL/day (5.5 mg of RYLGY and AYFYPEL)	6	−12 mm Hg in SBP	[29]
VY	A beverage enriched with sardine muscle hydrolysate	Randomized placebo-controlled trial, 29 subjects with mild hypertension.	2 × 100 mL (6 mg VY)	4	−9.3 mm Hg	[151]
	A vegetable drink	Randomized placebo-controlled trial, 63 subjects (male/female: 51/12) with mild hypertension.	195 g (0.5 g VY)	13	−7.6 mm Hg	[152]

## 5. General Conclusions

There is a tremendous global interest in promoting the use of food proteins/peptides as novel alternatives for present pharmaceutical therapeutics in the treatment and prevention of high blood pressure. In addition to exerting *in vivo* efficacy as the antihypertensive agent, food protein derived bioactive peptides can interact with the various blood pressure regulatory pathways, indicating their potential roles in controlling other pathologies related to the cardiovascular system, which may represent the advantage of using bioactive peptides as functional food/nutraceutical ingredients. However for many peptides, the actual mechanisms of action are not fully elucidated. Therefore, further research is required to identify the molecular targets of peptide action, which is important to establish the health promoting effects of these bioactive peptides. In this context, the use of advanced biochemical technologies such as proteomics, RNA sequencing, computational study with molecular docking and gene functional analysis are important to unlock the molecular mechanisms. Various clinical studies involving volunteers from different ethnic groups are also required to evaluate the ultimate efficacy and the pharmacokinetics of the active peptides. Furthermore, the safety of these bioactive peptides should also be evaluated before commercialization. Concomitant long-term research is also required to study the adverse or toxic effects associated with these active peptides. The translation of food derived bioactive peptide for human health improvements is an exciting scientific challenge, but simultaneously offers the opportunity for successful commercial applications. Lastly, various food materials with high protein content as well as the byproducts of the food processing industries could be used as a raw material for the industrial production of pharmaceutical grade bioactive peptides that may result in reduction in the production cost and also provides a sustainable and effective way of handling waste materials.

## Abbreviation

ACE 2: Angiotensin converting enzyme 2
ACE: Angiotensin I converting enzyme
Ang I: Angiotensin I
Ang II: Angiotensin II
APoE: Apolipoprotein E
BP: Blood pressure
cGMP: cyclic guanosine-monophosphate
CVDs: Cardiovascular diseases
DBP: Diastolic blood pressure
ECE: endothelin converting enzyme
ECs: Endothelial cells
EGFs: Epidermal growth factors
eNOS: Endothelial nitric oxide synthase
ET-1: Endothelin
GPCR: g-protein coupled receptor
ICAM-1: Intercellular adhesion molecule 1
IL: interleukin
iNOS: inducible nitric oxide synthase
KKS: Kallikrein kinin–system
MAP: Mean arterial pressure
MCP-1: Monocyte chemotactic protein-1
MMP: metalloproteinases
NADPH: nicotinamide adenine dinucleotide phosphate
NEP: neutral endopeptidase
NO: Nitric oxide
PGI2: Prostacyclin
RAS: Renin angiotensin system
ROS: Reactive oxygen species
SBP: Systolic blood pressure
sGC: soluble guanylyl cyclase
TNF-α: Tumor necrosis factor-α
VCAM-1: Vascular adhesion molecule-1
VSM: Vascular smooth muscle
